# Mixed pathologies and neural reserve: Implications of complexity for Alzheimer disease drug discovery

**DOI:** 10.1371/journal.pmed.1002256

**Published:** 2017-03-14

**Authors:** David A. Bennett

**Affiliations:** Rush Alzheimer’s Disease Center, Rush University Medical Center, Chicago, Illinois, United States of America

## Abstract

In a Perspective, David Bennett makes a case for neural reserve to be considered as a therapeutic endpoint in clinical trials for dementia.

Alzheimer disease (AD) is among the most common causes of dementia worldwide. Approved medications for the symptoms of AD dementia have marginal benefit, and no new therapies have been approved in more than a dozen years. There are no approved treatments for the prevention of AD dementia. The lack of robust therapeutics comes despite intense efforts by the public and private research community to discover them. There have been about 450 failed clinical trials on www.clinicaltrials.gov since the last drug approval by the Food and Drug Administration [1]. With changing demographics forecasting a marked increase in the number of persons with AD and other dementias worldwide, and with extraordinary attendant costs of care and toll on individuals and families, countries around the globe are investing substantial capital to stimulate basic and translational research in the hopes of developing new therapeutics for the treatment and prevention of AD and other dementia.

Clinical-pathologic findings, many from community-based studies that incorporate brain autopsy, are shedding light on the complexity of the AD dementia phenotype. This complexity, largely the result of mixed dementia and neural reserve, has important implications for clinical trial design and drug discovery.

## Mixed dementia

The pathologic hallmarks of AD are the extracellular deposits of the amyloid-β peptide and the intracellular accumulation of abnormally phosphorylated tau neurofibrillary tangles (NFT). Much is known about the metabolism of these proteins, and amyloid-β has been and remains the target of many therapeutic trials. The frequency of AD pathology increases markedly with age. Aging, however, is associated with the development of many common chronic diseases, including several brain diseases that affect cognition. Thus, the brains of older persons, many of whom have AD pathology, often exhibit macro- and/or microscopic infarctions, atherosclerosis, arteriolosclerosis, and white matter changes reflecting cerebrovascular disease, as well as tar DNA–binding protein 43 (TDP-43), hippocampal sclerosis, and neocortical Lewy bodies [2–5]. All of these pathologies can contribute to cognitive impairment and the AD dementia phenotype [6–8]. In fact, among older persons, who represent the greatest number of persons with the disease, mixed pathologies are the most common cause of AD dementia. Mixed pathologies are also very common in persons with mild cognitive impairment (MCI), including amnestic MCI, often thought to be relatively specific for MCI due to AD [9]. Some studies suggest that AD pathology accounts for the majority of dementia cases [10,11]. However, other studies suggest that the comorbid pathologies account for an equal amount of dementia and perhaps even more than AD, collectively [12].

Further, data suggest that the pathologies of these conditions account for less than half of the person-specific variance in change in cognition over multiple years prior to death [13]. It is highly likely that better methods to identify pathologies will be developed in the future—much like the relatively recent introduction of phospho-synuclein for visualizing Lewy bodies—and that new pathologies will be identified in the future, much like the relatively recent discovery of TDP-43. These future developments will reduce the unexplained variance. However, we also know that the brain, like other organs, has person-specific differences in its ability to protect itself from these pathologies—i.e., neural reserve.

## Neural reserve

The concept of reserve refers to the ability of a physiologic system to maintain function despite damage from injury or disease. All human physiologic systems exhibit reserve. In the context of the AD dementia syndrome, neural (or cognitive) reserve refers to the ability to maintain cognitive function despite the accumulation of the various pathologies that contribute to cognitive impairment. Nearly three decades ago, the first report of a series of persons without dementia who met pathologic criteria for AD was published [14]. It is now known that about a third of older persons without dementia or MCI meet pathologic criteria for AD, suggesting that many people are able to maintain excellent cognition despite the accumulation of brain pathology [15,16]. Amyloid-β cerebrospinal fluid (CSF) and positron emission tomography (PET) studies now suggest that AD pathology begins to accumulate years if not decades prior to the onset of clinical symptoms [17].

Among the most interesting things about neural reserve is that it may be amenable to lifestyle interventions. For example, there are many experiential and psychological risk factors for cognitive decline and AD dementia that do not appear to be related to any known brain pathology [18]. In other words, many factors can increase or decrease neural reserve agnostic to the underlying brain pathologies. Some studies have estimated that the number of AD dementia cases can be reduced by about a third through lifestyle interventions [19]. The interventions identified would likely reduce the burden of vascular disease, but others would operate through other, as yet unidentified mechanisms. Whether any would have a major effect on AD pathology itself seems unlikely [20–23]. Finally, we are now beginning to elucidate the cellular and molecular machinery underlying neural reserve [24–26].

## Implications of complexity for drug discovery

The data paint a picture of cognitive decline, MCI, and AD dementia resulting from a complex interaction between the accumulation of one or more brain pathologies in the context of a brain that is more or less resilient to these pathologies. This complexity has important implications for both clinical trial design and drug discovery.

Consider a drug that targets amyloid-β. Studies suggest that AD pathology accounts for about a third of the variance of cognitive decline if one includes the effects of both amyloid-β and NFT [7]. One can enrich a study for amyloid-β with PET scans or CSF amyloid-β, but the study still needs to be powered to affect only that portion of the cognitive trajectory associated with this pathology. It is not clear that most studies have explicitly powered their trials in this fashion.

Further, consider the sheer number of pathologies that contribute to AD dementia. Is it scalable to use a cocktail to target each one and to give these cocktails to older persons with aging kidneys and livers? Can health care systems absorb these costs, particularly in lower- and middle-income countries or even high-income countries with challenged economies and health systems? Currently, trials targeting a single molecular marker (i.e., amyloid-β) are expensive, requiring many costly PET scans.

By contrast, consider neural reserve as a therapeutic endpoint. There is no evolutionary pressure to create systems that protect the brain from any brain pathology of old age, let alone different systems that offer protection from different pathologies. Thus, finding that myriad factors alter the trajectory of cognitive decline agnostic to underlying brain pathologies is expected. A hypothetical therapeutic agent that targets neural reserve could be used to offset any and likely all common brain pathologies that alter cognition. How one would design such a clinical trial is a challenge that would need to be vetted with regulatory agencies. The simplest approach, assuming a relatively safe agent, would be a large longitudinal study of cognitive decline among people at slightly elevated risk of cognitive decline (e.g., over age 74 with cognitive complaints). Depending on the anticipated effect size, such a study would likely need a minimum of a few thousand subjects followed for at least four years. In such a design, one would let the randomization distribute the common brain pathologies relatively equally between the study arms and simply use the rate of cognitive decline as the endpoint. However, should regulatory agencies require proof that the mechanism of action is reserve and/or require that the trial demonstrate target engagement, study designs could become very complex and expensive. Although many issues remain to be addressed, neural reserve offers a new paradigm for approaching the treatment and prevention of AD and indeed all dementia syndromes.

## Abbreviations

AD: Alzheimer disease
CSF: cerebrospinal fluid
MCI: mild cognitive impairment
NFT: neurofibrillary tangle
PET: positron emission tomography
TDP-43: tar DNA–binding protein 43
